# Targeted Methotrexate Treatment in Cesarean Scar Pregnancy: A Case Report and Literature Review

**DOI:** 10.1155/crog/4653003

**Published:** 2026-11-13

**Authors:** Marta Adamczyk, Helena Limowska, Aneta Zwierzchowska, Piotr Merks, Ewa Barcz

**Affiliations:** ^1^ Collegium Medicum Cardinal Stefan Wyszyński University Warsaw Poland; ^2^ Department of Obstetrics and Gynecology Międzylesie Specialist Hospital Warsaw Poland; ^3^ Trade Union of Pharmacy Workers (ZZPF) Warsaw Poland

**Keywords:** cesarean scar pregnancy, cesarean section, ectopic pregnancy, methotrexate, vaginal bleeding

## Abstract

**Introduction:**

Cesarean scar pregnancy (CSP) represents a rare form of ectopic pregnancy, though its incidence is expected to rise in countries with rising rates of deliveries by cesarean section (CS). Delayed diagnosis and treatment can result in uterine rupture and hemorrhage. Diagnosis is based on transvaginal ultrasonography (TVUS). No standardized treatment protocol for CSP has been established; however, local ultrasound‐guided methotrexate (MTX) injection into the gestational sac (GS) is widely recognized as a highly effective therapy.

**Case Report:**

A 38‐year‐old woman with a history of CS 2 years prior was admitted to the hospital due to moderately heavy vaginal bleeding that had started 4 days before. Her general condition was assessed as stable. The last menstrual period occurred 6 weeks and 2 days earlier. A *β*‐hCG test performed on presentation showed a level of 10,775 mIU/mL. TVUS revealed a GS measuring 8.6 mm located within the CS scar, containing a yolk sac and an embryo with a CRL of 2.8 mm. Cardiac activity had not been detected. A diagnosis of CSP was made. Other laboratory tests showed no abnormalities. On the following day, the *β*‐hCG level had increased to 13,001 mIU/mL. Under TVUS guidance, 80 mg of MTX was injected into the GS. Following days showed a gradual decline in *β*‐hCG levels, reaching 927.77 mIU/mL on the third day postprocedure. A follow‐up TVUS revealed no visible GS. The patient remained in stable condition and was discharged from the hospital. On Day 10 postprocedure, the *β*‐hCG level had decreased to 62.04 mIU/mL. Aside from mild uterine bleeding, the patient reported no other complaints.

**Conclusions:**

In the presented case, early detection followed by timely ultrasound‐guided MTX injection into the GS achieved complete resolution without requiring additional interventions, highlighting its potential as a safe and effective conservative approach when CSP is diagnosed early.

## 1. Introduction

Cesarean scar pregnancy (CSP) is no longer an exceedingly rare clinical entity; as reported by Litwicka and Greco [1], it now accounts for approximately 6.1% of all ectopic pregnancies in patients with a history of at least one prior CS. It occurs when the GS implants within the myometrial defect of a previous uterine scar. The prevalence of CSP has increased over the past 2 decades, paralleling the global rise in cesarean delivery rates [2].

Approximately one‐third of cases are asymptomatic, whereas another one‐third present with painless vaginal bleeding. In many instances, the diagnosis is established during routine first‐trimester ultrasound examination. Early diagnosis and prompt management are crucial to prevent serious complications, including massive hemorrhage, uterine rupture, abnormal placentation (placenta accreta spectrum [PAS]), and maternal morbidity and mortality [3].

As highlighted by Hameed et al. [4] in their comprehensive review on minimally invasive techniques, there is still no single global consensus on a standardized treatment protocol for CSP. However, local ultrasound‐guided MTX injection is widely recognized as a highly effective and safe first‐line modality for early, unruptured cases in stable patients. Although minimally invasive surgical options like laparoscopy offer the advantage of simultaneous scar repair, our case confirms that ultrasound‐guided local medical management alone can achieve complete resolution and successfully preserve long‐term fertility without surgical morbidity. Existing recommendations from professional societies are primarily based on case series, systematic reviews, and a limited number of randomized studies [5]. The small sample sizes and heterogeneity of available studies limit the strength of the evidence and allow for variability related to clinical expertise and institutional experience [3].

Currently available treatment options include systemic or local MTX administration (including intragestational injection), hysteroscopy (HSC), dilation and curettage (D&C), uterine aspiration, uterine artery embolization (UAE), laparoscopy, laparotomy, direct potassium chloride (KCl) injection, needle‐guided sac decompression, high‐intensity focused ultrasound (HIFU), various combined approaches, and expectant management [3].

If left untreated, CSP may progress to PAS, particularly in women with a history of cesarean delivery and coexisting placenta previa. The cesarean scar niche may create a localized environment that facilitates abnormal trophoblastic invasion. Disruption of the endometrial and myometrial layers further predisposes to deep implantation [6]. The endogenic type of CSP carries a lower risk of progression to PAS, whereas approximately 50% of exogenic cases may evolve into PAS. Type III CSP may predispose to placenta percreta, increasing the risk of severe hemorrhage and the need for hysterectomy [6].

Herein, we present a case of CSP successfully treated with local MTX administration, followed by a review of the current literature regarding this and other therapeutic approaches for this potentially life‐threatening early pregnancy complication.

## 2. Case Presentation

A 38‐year‐old woman (Gravida 2, Para 1) with B Rh‐positive blood type presented to the emergency department (ED) with painless vaginal bleeding that had persisted for 4 days. She had undergone a CS 2 years earlier. Her last menstrual period had occurred 6 weeks and 2 days prior to presentation. She had no history of other surgeries and no comorbidities.

At an outpatient visit 4 days before admission, her serum *β*‐hCG level was 1442.6 mIU/mL. TVUS performed at that time did not reveal a GS within the uterine cavity.

The day before presentation to the ED, the serum *β*‐hCG level was 6213.8 mIU/mL, and on admission it had increased to 10,775 mIU/mL. Complete blood count results were within normal limits. TVUS demonstrated an empty uterine cavity with an endometrial thickness of 10 mm and a GS containing a yolk sac located within the cesarean scar niche (Figures 1, 2, and 3). The mean GS diameter was 8.6 mm, and the crown–rump length (CRL) measured 2.8 mm, corresponding to a gestational age of 5 weeks and 5 days. No adnexal abnormalities were identified.

**Figure 1:**
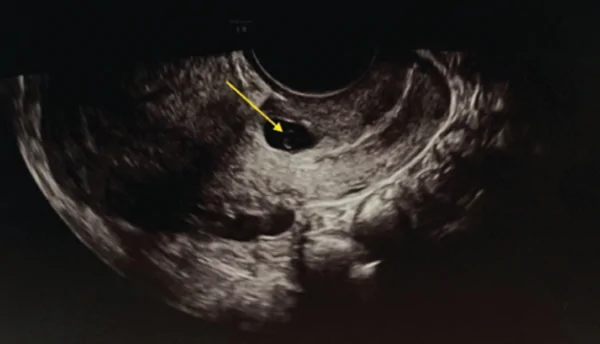
GS in the CS scar had a diameter of 8.6 mm, and the CRL = 2.8 mm, corresponding to a gestational age of 5 weeks and 5 days.

**Figure 2:**
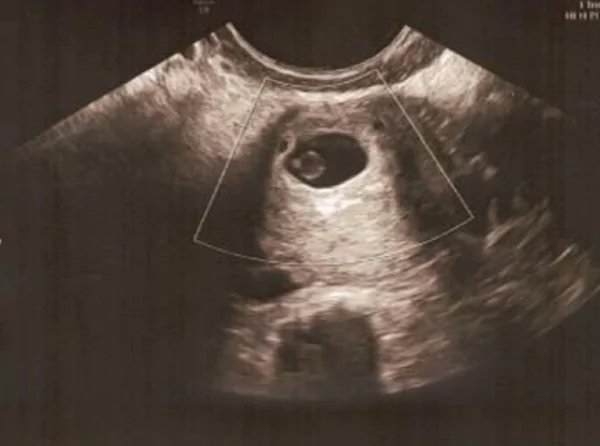
The GS is located within the anterior lower uterine segment with marked thinning of the myometrium.

**Figure 3:**
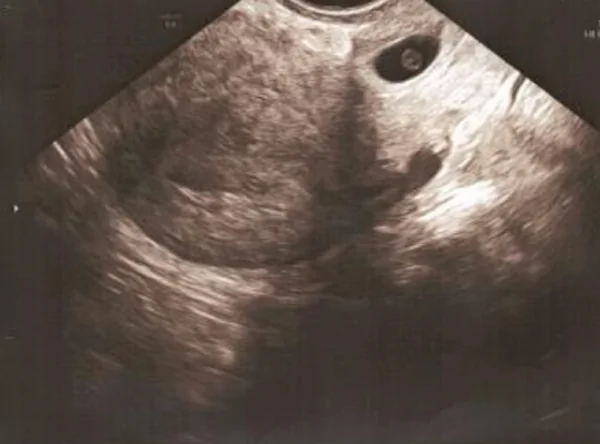
TVUS showing an empty uterine cavity and a low‐lying GS embedded in the anterior uterine wall at the CS scar.

On the following day, the *β*‐hCG level increased further to 13,001 mIU/mL. The patient was thoroughly counseled regarding the severe risks associated with CSP, including uterine rupture, catastrophic hemorrhage, and the potential need for an emergency hysterectomy. The available management options, including surgical resection (laparoscopy or HSC), systemic multidose methotrexate, and local ultrasound‐guided treatment were discussed. Given the patient′s stable hemodynamic status, the desire to preserve future fertility, and the thin myometrium at the scar site, a minimally invasive approach was favored. The patient was counseled regarding the risks associated with CSP and the available management options. She consented to local MTX treatment. The intervention was performed under short intravenous anesthesia. A TVUS probe equipped with a sterile needle guide was introduced. Under continuous real‐time ultrasound monitoring, the GS within the cesarean scar niche was clearly visualized, ensuring the bladder wall was safely bypassed. A 16‐gauge, 30‐cm single‐lumen aspiration needle was advanced transvaginally through the anterior vaginal fornix directly into the center of the GS. The total dose of 80 mg of MTX was administered. To ensure even and comprehensive infiltration of the gestational niche and the surrounding trophoblastic tissue, the total dose was divided into four consecutive 20‐mg injections delivered circumferentially into the trophoblast and the sac cavity during this single session. Upon needle withdrawal, color Doppler imaging was utilized to confirm the absence of active bleeding or hematoma formation at the vaginal puncture site and within the scar area. The total procedure time was 55 min, and the estimated blood loss was approximately 15 mL. No perioperative antibiotic prophylaxis was administered.

Three days after the procedure, the patient remained in good clinical condition and reported only mild vaginal bleeding. Ultrasound examination demonstrated resolution of the GS. The serum *β*‐hCG level decreased to 927.77 mIU/mL, and the patient was discharged in stable condition.

At follow‐up 10 days after treatment, the patient reported moderate vaginal bleeding without additional symptoms. TVUS confirmed an empty uterine cavity with a thin endometrium (Figure 4). No pathological masses or abnormalities were detected in the adnexa. However, within the cesarean scar niche, a well‐demarcated residual lesion measuring 9.4 × 9.5 mm without detectable vascular flow on Doppler imaging was observed. As the *β*‐hCG level had further declined to 62.04 mIU/mL, no additional therapeutic intervention was required. Given the downward trend of *β*‐hCG and the absence of active vascularization, expectant management with ongoing outpatient monitoring was continued, and the symptoms resolved spontaneously.

**Figure 4:**
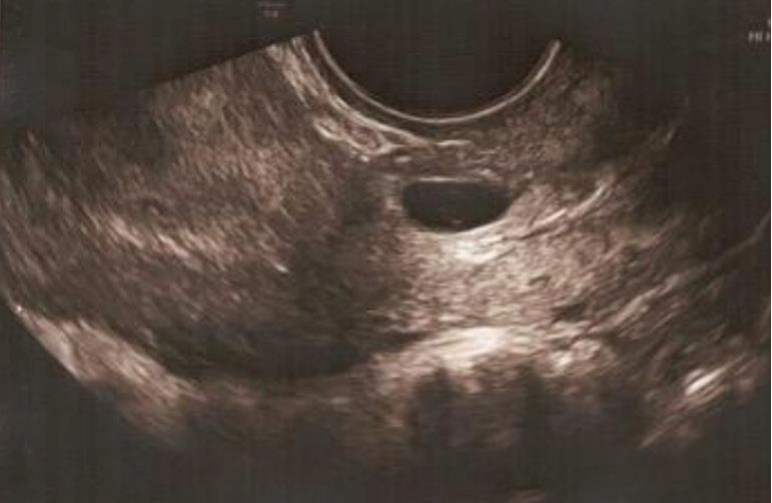
Well‐defined, anechoic residual lesion measuring 9.4 × 9.5 mm located in the CS scar after local MTX injection. The sac appears regular with no visible embryonic echoes or cardiac activity, consistent with early postprocedure involution.

One year later the patient conceived spontaneously. The subsequent pregnancy progressed without any maternal or fetal complications. Because of her obstetric history, she underwent a scheduled, elective CS at term (39 weeks of gestation), resulting in the delivery of a healthy newborn. No signs of abnormal placental invasion (such as PAS) or uterine scar dehiscence were noted intraoperatively.

Written informed consent was obtained from the patient for the publication of this case report and any accompanying images.

## 3. Discussion

CSP is a rare but increasingly recognized complication of CS, with an estimated incidence ranging from 1 in 1800 to 1 in 2656 pregnancies [3]. Awareness of this long‐term complication is crucial, as delayed diagnosis can result in severe maternal morbidity.

### 3.1. Pathophysiology

CSP occurs when a GS implants within a myometrial defect of a previous CS scar. Fibrosis within scar tissue may lead to small dehiscences, and the thicker the hysterotomy scar, the higher the risk of disrupted healing and defect formation [3]. Several mechanisms contribute to implantation on a cesarean scar, including invasion of the myometrium through a microtubular tract connecting the scar to the endometrial canal. Scar tissue often exhibits disrupted myofiber organization, inflammation, elastosis, edema, apoptosis, and reduced smooth muscle density, facilitating the invasion of extravillous trophoblastic cells beyond the inner myometrium to reach outer myometrial vessels [6]. Additionally, hypoxia within the scar niche may stimulate the trophoblast to penetrate deeply into the myometrium [6].

### 3.2. Clinical Presentation

Scant vaginal bleeding is the most characteristic symptom of CSP, in contrast to cervical pregnancy or miscarriage, where bleeding is usually more profuse. Mild lower abdominal pain may occur; however, up to one‐third of patients can remain asymptomatic [7].

### 3.3. Diagnosis and Imaging

TVUS is the first‐line imaging modality for CSP, whereas MRI is reserved for ambiguous cases [8]. Sonographic criteria include the following:

• Empty uterine cavity and endocervical canal.
• Placenta and/or blastocyst embedded in the cesarean scar.
• Triangular, oval, or rounded GS within the scar.
• Thin or absent myometrial layer between the GS and bladder.
• Rich vascularity at the scar site.
• Presence of a fetal pole and/or yolk sac, with or without cardiac activity [3].

CSP can be misdiagnosed as cervical pregnancy or spontaneous miscarriage. Accurate differentiation requires careful evaluation of GS size, shape, and location. In our case, US clearly demonstrated an empty uterine cavity and cervical canal, with the GS distinctly positioned within the scar niche.

### 3.4. Classification

Salvo et al. [6] categorize CSP as endogenic (“on the scar”) or exogenic (“in the niche”). Endogenic CSP grows toward the uterine cavity, typically with myometrial thickness > 3 mm, whereas exogenic CSP originates from a deeply implanted blastocyst, often with myometrial thickness < 3 mm, and may extend toward the bladder or abdominal cavity. Endogenic CSP carries a high risk of hemorrhage and complications, including PAS, whereas untreated exogenic CSP frequently progresses to PAS, often necessitating hysterectomy [9].

### 3.5. *β*‐hCG Dynamics

Serial serum *β*‐hCG measurements can aid in diagnosis. Generally, ectopic pregnancies demonstrate a slower increase in *β*‐hCG compared with intrauterine pregnancies. Even so, CSP may mimic a viable intrauterine pregnancy, similar to heterotopic pregnancies [5]. Our patient′s initial *β*‐hCG trend initially suggested a viable intrauterine pregnancy.

### 3.6. Treatment Approaches

Multiple treatment modalities exist for CSP, encompassing medical management (systemic or local MTX, KCl injection), minimally invasive and interventional procedures (HSC, UAE, and HIFU), surgical approaches (D&C, uterine aspiration, laparoscopy, and laparotomy), and various combination therapies [3].

MTX can be administered systemically (typically 50 mg/m^2^) or locally into the GS (commonly 1 mg/kg or 50 mg/m^2^), as a single or multidose regimen with folinic acid [10]. Koch et al. [11] reported no significant differences in bleeding or pain between systemic and local MTX, although *β*‐hCG clearance was 29.2 days longer with local MTX. Conversely, Kim et al. [12] suggested that local MTX may provide superior outcomes due to reduced blood flow in the scar niche, which facilitates local drug accumulation [3].

Common side effects include vaginal spotting, nausea, vomiting, diarrhea, and mild abdominal pain. Patients should avoid folic acid supplements, NSAIDs, alcohol, opioids, and activities that could mask symptoms of rupture. Contraception is advised for 3 months posttreatment due to MTX teratogenicity. Rising *β*‐hCG levels or clinical instability during treatment necessitate surgical intervention. Emerging biomarkers such as serum CPK, red cell distribution width, mean platelet volume, and neutrophil‐to‐lymphocyte ratio may help predict treatment success [13].

Local MTX injection is most effective under certain conditions:

• Gestational age < 8 weeks.
• Absence of fetal cardiac activity.
• Myometrial thickness < 2 mm.
• *β*‐hCG < 5000 mIU/mL [14].

Although baseline *β*‐hCG levels below 5000 mIU/mL are widely regarded in the literature as an optimal predictor for the success of MTX therapy, our case demonstrates that successful conservative management with local MTX injection remains feasible and effective even at significantly higher, increasing titers (exceeding 10,000 mIU/mL). Therefore, although lower *β*‐hCG values are associated with higher success rates, elevated levels should not be viewed as an absolute contraindication to local MTX administration, provided that careful patient selection and close monitoring are maintained. The general clinical principles, eligibility criteria, and established systemic dosing regimens for methotrexate administration are summarized in Table 1.

**Table 1:** Methotrexate therapy [13, 15–17].

Indications:	• Hemodynamically stable patient
	• No evidence of rupture
	• Willingness and ability for close follow‐up
	• Small ectopic size (< 3.5–4 cm)
	• Lower initial *β*‐hCG (< 5000 mIU/mL preferred)
	• Absence of fetal cardiac activity preferred
Absolute contraindications:	• Intrauterine pregnancy
	• Breastfeeding
	• Hypersensitivity to MTX
	• Moderate‐severe anemia, leukopenia, or thrombocytopenia
	• Significant hepatic or renal impairment
	• Active peptic ulcer disease or pulmonary disease
	• Immunodeficiency
	• Evidence of rupture or hemodynamic instability
Dosing protocols:	• Single‐dose: 50 mg/m^2^ IM, *β*‐hCG check on Days 4 and 7; (≥15*%*drop = success, otherwise second dose)
	• Multiple‐dose: 1 mg/kg IM on Days 0, 2, 4, and 6 with folinic acid rescue on alternate days; (higher risk of side effects, less commonly used)
Monitoring posttreatment:	• *β*‐hCG on Days 4 and 7, then weekly until undetectable
	• Monitor symptoms and vital signs
	• If *β*‐hCG plateaus or rises, consider a repeat injection or secondary surgical intervention
Common side effects:	• Abdominal cramping/pain
	• Vaginal bleeding or spotting
	• Nausea, vomiting, and indigestion
	• Oral stomatitis
	• Fatigue
Rare side effects:	• Elevated liver enzymes
	• Bone marrow suppression
	• Dermatitis (sun sensitivity)
	• Pneumonitis
	• Alopecia
Predictors of success:	• Initial *β*‐hCG < 5000 mIU/mL (success > 90% when < 1000 mIU/mL)
	• Absence of embryonic cardiac activity and smaller gestational sac size

The literature reports success rates of 65%–75% for local MTX and 71%–80% for systemic MTX [3, 18–20]. Combination therapies, involving multiple MTX injections via systemic and local routes, have demonstrated variable success, with additional interventions sometimes required (as summarized in Table 2). In our case, local MTX treatment was effective, with no adverse effects and no need for further interventions.

**Table 2:** Methotrexate treatment in the studies.

Study	Patients	MTX administration	Success rate	Complications
[3]	96 overall (45 treated with MTX)	Local intragestational MTX	73.9% (single injection); 88.5% (cumulative after secondary injection)	Not reported
[18]	9	Local intragestational MTX under US guidance	75%	Minimal blood loss; no major complications; one patient required UAE within 1 month
[19]	5290 overall (123 treated with MTX)	Triple protocol: intragestational (fetal heart and placental tissue) plus systemic MTX	50.4%	Secondary interventions: laparoscopy, open surgery, hysterectomy. Adverse effects: bleeding, fever, elevated liver enzyme levels
[20]	72 with early ectopic pregnancy (including CSP, 7%)	Systemic MTX	Tubal ectopic: 73% (single dose); persistent pregnancy of unknown location: 88%; CSP: 100% (no additional intervention needed)	Not reported

*Note:* Data are presented as numbers of cases or percentages as reported in the respective studies. Cumulative success rate reflects resolution of ectopic gestation following one or more doses of MTX without conversion to surgical or alternative interventional procedures.

The enhanced efficacy of local injection may be explained by the fact that the GS is encased in fibrous scar tissue rather than normal myometrium, which can impede the penetration of the drug from the bloodstream into the GS. Furthermore, the reduced blood flow to the scar niche facilitates the accumulation of MTX within the sac, enhancing its local effectiveness [3].

According to the Society for Maternal–Fetal Medicine (SMFM), intragestational KCl injection may be preferable in cases of heterotopic CSP due to the teratogenic effects of MTX [3]. Salari et al. presented two cases of CSP treated with combined TVUS‐guided KCl injection (20 mEq/10 mL, 15% w/v) and four doses of intravenous MTX (1 mg/kg). Both patients, aged 31 and 34, had prior cesarean deliveries. Their gestational sacs contained live embryos, and initial *β*‐hCG levels were 50,099 and 297,969 mIU/mL, respectively. The treatment was well tolerated, and *β*‐hCG levels declined over the following weeks [17].

The therapeutic efficacy of D&C, performed with or without ultrasound guidance, depends heavily on specific anatomical characteristics rather than technical variation alone. Success rates correlate strongly with CSP morphology, dropping from 64.3% in Type I to only 14.3% in Type II lesions [21]. Furthermore, residual myometrial thickness is a critical determinant of procedural safety and outcome, yielding a 97.7% success rate when the scar exceeds 3 mm compared with only 50.0% in thinner defects [22].

UAE is considered a reasonable treatment option for CSP, particularly for reducing the risk of hemorrhage [23]. A single‐center study published in 2025 compared UAE combined with US‐guided D&C, with or without systemic MTX. Both protocols achieved similar success rates, but the addition of MTX shortened the time to *β*‐hCG normalization and reduced the length of hospital stay [24].

Multimodal protocols are frequently utilized to optimize outcomes in challenging presentations. Common regimens include systemic MTX combined with UAE, or local/systemic MTX paired with KCl injection. In cases with confirmed embryonic cardiac activity, intracardiac KCl followed by intragestational MTX has proven reliable in arresting embryonic heart tones and inducing gestational sac collapse [25]. Similarly, combining systemic MTX with vacuum aspiration has demonstrated superior efficacy compared with MTX with conventional curettage [26].

Surgical intervention remains an important organ‐preserving or definitive option, with laparoscopy offering distinct advantages over laparotomy, including reduced intraoperative blood loss and shorter hospitalization [27]. Laparoscopic resection paired with temporary internal iliac or uterine artery occlusion provides optimal perioperative parameters (lower transfusion rates and faster recovery) without compromising *β*‐hCG clearance or increasing reoperation rates [27].

When patients decline termination and opt to continue gestation despite substantial risks, rigorous serial ultrasound surveillance is imperative to detect morbid PAS early. Although isolated cases of CSP progressing to live full‐term delivery have been documented, maternal morbidity remains severe: Nearly one‐third require peripartum hysterectomy [5], and more than half of those managed expectantly ultimately require emergent surgical intervention [28].

## 4. Conclusion

Despite the lack of clear guidelines and the variety of available therapeutic options, based on the case we described and a comprehensive review of the literature, it can be concluded that early intragestational MTX administration can be an effective organ‐preserving option capable of achieving complete resolution without adjunct procedures. Nonetheless, given that these findings are drawn from an individual case, they should be viewed as an encouraging observation rather than a generalized treatment protocol.

## Author Contributions

Marta Adamczyk: investigation, writing—original draft, validation, writing—review and editing, resources, data curation, visualization. Helena Limowska: writing—original draft, data curation, investigation, resources. Aneta Zwierzchowska: conceptualization, methodology, data curation, validation, supervision, investigation, formal analysis, project administration. Piotr Merks: funding acquisition, supervision. Ewa Barcz: conceptualization, data curation, resources, supervision, project administration.

## Funding

The publication was funded by the Trade Union of Pharmacy Workers (ZZPF).

## Disclosure

All authors have read and agreed to the published version of the manuscript.

## Conflicts of Interest

The authors declare no conflicts of interest.

## Data Availability

The data that support the findings of this study are available from the corresponding author upon reasonable request.
